# A Simple Method for Evaluating the Bioactive Phenolic Compounds’ Presence in Brazilian Craft Beers

**DOI:** 10.3390/molecules26164716

**Published:** 2021-08-04

**Authors:** Marcelo Coelho Silva, Jeancarlo Pereira dos Anjos, Lilian Lefol Nani Guarieiro, Bruna A. Souza Machado

**Affiliations:** 1 Centro Universitário SENAI CIMATEC, Avenida Orlando Gomes, 1845-Piatã, Salvador 41650-010, BA, Brazil; celo12br@hotmail.com (M.C.S.); lilian.guarieiro@fieb.org.br (L.L.N.G.); brunam@fieb.org.br (B.A.S.M.); 2 INCT de Energia e Ambiente, UFBA, Salvador 40170-290, BA, Brazil

**Keywords:** craft beer, beverage, phenolic compounds, HPLC-DAD

## Abstract

There are a significant number of analytical methodologies employing different techniques to determine phenolic compounds in beverages. However, these methods employ long sample preparation processes and great time consumption. The aim of this paper was the development of a simple method for evaluating the phenolic compounds’ presence in Brazilian craft beers without a previous extraction step. Catechin, caffeic acid, epicatechin, *p*-coumaric acid, hydrated rutin, *trans*-ferulic acid, quercetin, kaempferol, and formononetin were analyzed in fifteen different craft beers. The method showed good linearity (R^2^ ≥ 0.9966). The limit of detection ranged from 0.08 to 0.83 mg L^−1^, and limits of quantification were between 0.27 and 2.78 mg L^−1^. The method showed a satisfactory precision (RSD ≤ 16.2%). A good accuracy was obtained by the proposed method for all phenolic compounds in craft beer (68.6% ˂ accuracy ˂ 112%). Catechin showed higher concentrations (up to 124.8 mg L^−1^) in the samples, followed by epicatechin (up to 51.1 mg L^−1^) and caffeic acid (up to 8.13 mg L^−1^). Rutin and formononetin were observed in all analyzed samples (0.52 mg L^−1^ to 2.40 mg L^−1^), and kaempferol was less present in the samples. The presence of plant origin products was determinant for the occurrence of the highest concentrations of phenolic compounds in Brazilian craft beers.

## 1. Introduction

Beer is a widespread drink on a world scale, and therefore its consumption is extremely broad and appreciated by a wide audience. It is the third most consumed beverage around the world and the first among the alcoholic beverages [1,2]. Brazil is the third largest producer in the world, behind only China and the United States. Beer is the most consumed alcoholic drink in Brazil, with an estimated annual consumption of approximately 82 L/inhabitant [3].

Beer is produced by the alcoholic fermentation from raw materials including barley malt and grains, hops, water, and yeast. Different combinations of ingredients in different proportions as well as the use of varied methods of production culminate in a chemically complex product, resulting in both different types and styles of beers [4,5,6]. Riu-Aumatell et al. (2014) [4] mentioned that more than 1000 compounds have already been identified in beer, either originating from raw material or forming during processing.

Beer consists of a mixture of several chemical substances among which are carbohydrates, minerals (potassium, magnesium), vitamins (niacin, riboflavin, folate, cobalamin, pyridoxine), and amino acids. In addition, beer has a significant amount of phenolic compounds (considered as bioactive substances, mainly due to their antioxidant properties), which originate from the malt (70–80%) and hops used in the production of the beverage. Hydroxybenzoic acids, cinnamic acids (such as ferulic acid), and flavonols are the main phenolic compounds found in beers [7,8,9]. The phenolic compounds predominant in beer are ferulic, *p*-coumaric, vanillic, and protocatechuic acids and small amounts of catechin, *p*-hydroxybenzoic, chlorogenic, caffeic, and sinapic acids [10,11,12].

The polyphenolic composition of beers can be considered as one quality indicator of the beverage processing and marketing. The type and quantity of phenolic compounds influence the taste, color, foam, beer turbidity, bitterness, colloidal, and sensory properties besides of stability and shelf-life of beer [2,13,14].

In addition to their important role in the sensory characteristics of the beverage, phenolic compounds are excellent electron donors, acting as antioxidant preservatives in foods and radical scavengers in physiological systems [1]. Several studies have associated the consumption of foods and beverages rich in phenolic compounds (including the moderate consumption of alcoholic beverages or those with a low ethanol content) to the health benefits of consumers, such as prevention of cardiovascular diseases and certain types of cancer and the prevention of other diseases related to aging [15,16,17,18,19,20,21]. Although the serious harms of high alcohol intake are known, moderate beer consumption is believed to be associated with protective cardiovascular function and reduction in the development of neurodegenerative diseases [2].

In addition to the popularly known industrial beers, the production and consumption of craft beers have grown significantly in Brazil and the world in recent years. The increase in the number of microbreweries has become evident, with an increasing variety of type of craft beers available in markets and pubs [22]. No single definition for craft beer has been adopted due to the diversities among countries and their historically different traditions in beer brewing [23]. However, craft beers are those that are made with small quantities of natural products in their production and are modified in a particular way by each producer until they have specific organoleptic properties [24].

Craft beers produced with the addition of fruits, spices, or natural foods during the fermentation process have become very popular throughout the world, responding to consumer requests for new gustatory, olfactory, and visual stimuli. In this way, the addition of natural products to the beverage can increase the content of bioactive compounds and the oxidative stability of the beverage [11,25]. Despite that beer is considered a beverage rich in bioactive and antioxidant substances, such as phenolic compounds, a great amount of information can still be explored regarding the presence of these compounds in craft beers due to the diversity of substances present in the beverage [22].

Analytical methodologies employing different techniques have been cited in the literature to determine bioactive compounds, including phenolic compounds in beverages [26,27,28]. However, these methods employ long sample preparation processes and great time consumption. Wannenmacher et al. (2018) [29] related several papers that evaluated the presence of phenolic compounds in beers. Almost all the studies have employed previous extraction or preconcentration methods of the analytes in the samples. Pretreatment techniques, such as liquid–liquid extraction, have been extensively used in the determination of phenolic compounds in beers. However, there is a great lack in the direct analysis of beer samples for the determination of phenolic compounds. The advantages of direct analysis of beverage samples include the reduction in experimental errors related to sample handling, shorter analysis time, costs reduction due to lower input use, and little or no generation of organic solvents wastes on sample-preparation step. Although analysis using HPLC still generates a significant amount of residues from column elution, the sample-preparation step could be in accordance with principles established by the Green Analytical Chemistry, related to the characteristics mentioned above [30,31].

Thus, this paper aims to propose a simple method for evaluating the phenolic compounds presence (catechin, caffeic acid, epicatechin, *p*-coumaric acid, hydrated rutin, *trans*-ferulic acid, quercetin, kaempferol, and formononetin) in Brazilian craft beers, using high-performance liquid chromatography with diode array detection (HPLC-DAD) without a previous extraction step of the analytes.

## 2. Results

### 2.1. Chromatographic Profile of the Phenolic Compounds in Craft Beers

**Figure 1:**
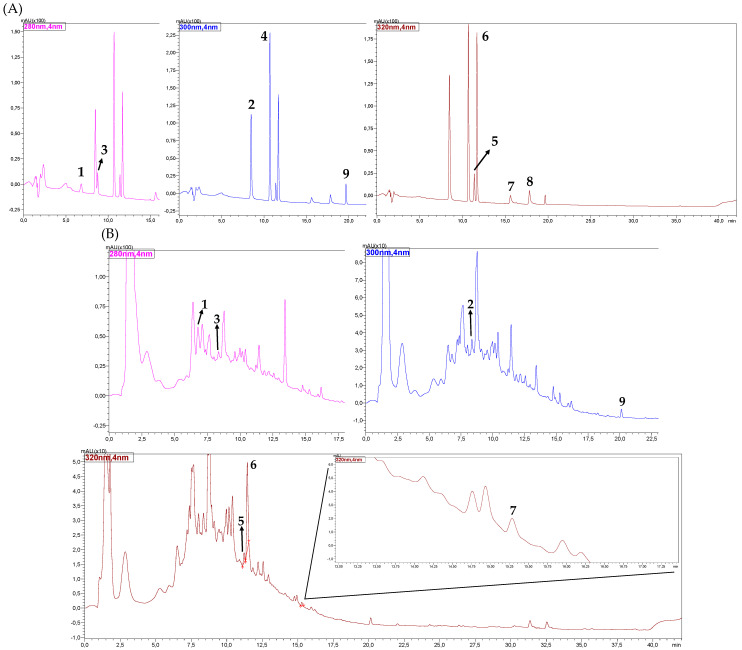
Chromatograms obtained by HPLC-DAD for the standard solution of the phenolic compounds at the concentration of 10 mg L^−1^ (**A**) and one sample of Brazilian craft beer (**B**). Identification of the peaks: 1. catechin; 2. caffeic acid; 3. epicatechin; 4. *p*-coumaric acid; 5. hydrated rutin; 6. *trans*-ferulic acid; 7. quercetin; 8. kaempferol; 9. formononetin (pink = 280 nm; blue = 300 nm; and brown = 320 nm).

Figure 1 shows the chromatograms relative to a standard solution of the phenolic compounds and one of the samples of craft beers obtained after the tests to optimize the chromatographic separation of the analytes.

In the Supplementary Material (Figure S3), it is possible to verify the chromatogram of the sample shown in Figure 1B with the superposition of the wavelengths used for the analysis of phenolic compounds in Brazilian craft beers.

### 2.2. Validation of the Method

#### 2.2.1. Selectivity

**Figure 2:**
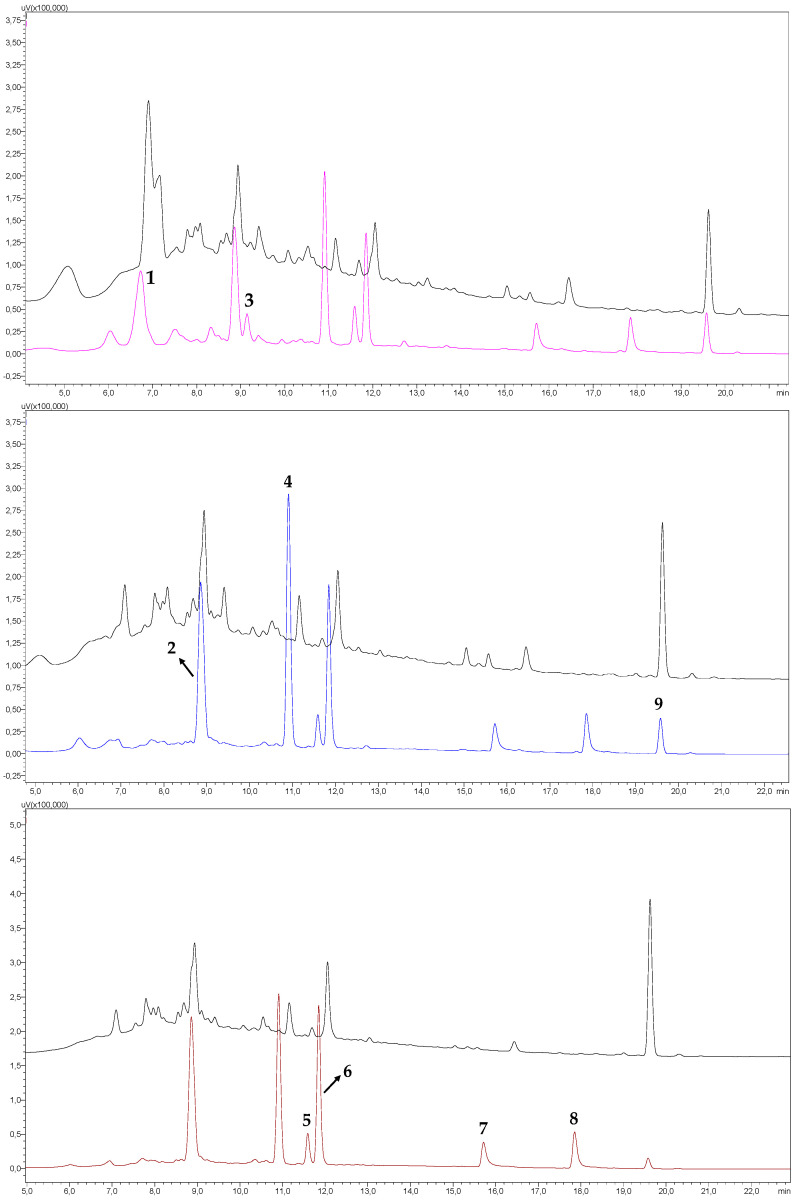
Chromatogram of the selectivity test, obtained by HPLC-DAD, containing one of the analyzed craft beer samples (Black line) and the same sample added with a standard solution of phenolic compounds (12.5 mg L^−1^) (pink = 280 nm; blue = 300 nm; and brown = 320 nm). Identification of the peaks: 1. catechin; 2. caffeic acid; 3. epicatechin; 4. *p*-coumaric acid; 5. hydrated rutin; 6. *trans*-ferulic acid; 7. quercetin; 8. kaempferol; 9. formononetin.

Under the chromatographic conditions employed, it was observed that the samples showed no interfering substances in the retention times of the phenolic compounds analyzed. The selectivity was verified by the comparison of the chromatogram of a sample without the addition of the phenolic compounds standard and of the same sample fortified with the standards of the compounds analyzed at a concentration of 12.5 mg L^−1^ for each analyte (Figure 2), thus confirming the selectivity of the analytical method.

#### 2.2.2. Linearity

After the construction of the analytical curves of the phenolic compounds, we obtained determination coefficients (R^2^) ranging from 0.9966 to 0.9999 (Table 1), showing the strong linear correlation between the concentration of the analyzed compounds and the areas of the chromatographic peaks, allowing the quantification of the phenolic compounds in samples of Brazilian craft beers.

#### 2.2.3. Limits of Detection and Quantification

After the optimization of the chromatographic conditions, different wavelengths were used to detect the respective phenolic compounds analyzed. Thus, limits of detection (LOD) varied from 0.08 to 0.83 mg L^−1^, and limits of quantification (LOQ) were between 0.27 and 2.78 mg L^−1^ (Table 1), certifying the good sensitivity of the proposed method. It is noteworthy that a good sensitivity of the method was obtained without the use of extraction and preconcentration procedures of the analytes.

#### 2.2.4. Precision

Table 2 shows the relative standard deviations (RSD) values obtained for the intraday precision tests of the analytical method.

In according to Thompson et al. (2002) [32], depending on the degree of complexity of the samples, RSD of up to 20% is accepted. Samples of craft beers can be considered complex since they have a great amount of chemical substances in their composition besides the phenolic composition studied in our work. For this reason, many studies use techniques of extraction, preconcentration, or clean-up for analyzing phenolic compounds in beers [5,10,29]. In our work, even without the use of these techniques, the coefficient of variation values were lower than 20% for all phenolic compounds in three concentration levels. In this way, the method showed a satisfactory precision for the phenolic compounds analyzed in Brazilian craft beers.

#### 2.2.5. Accuracy

The accuracy of the method was evaluated by means of standard addition tests at three levels of concentration in a sample of craft beer. Table 3 shows the results obtained in the accuracy tests of the method for the determination of phenolic compounds in Brazilian craft beer. It is evident that a good accuracy was obtained by the proposed method for all phenolic compounds in craft beer (68.6% ˂ accuracy ˂ 112%), whose values fall within recommended limits. In according to Thompson et al. (2002) [32], the acceptable ranges in recovery tests could be between 70% and 120%. However, depending of analytical method and samples complexity, this range could be of 50% to 120%.

### 2.3. Application of the Method in Real Samples

After the validation step, the proposed method was applied in real samples of Brazilian craft beers. The phenolic composition of the samples presented a varied concentration of the compounds analyzed according to the type of beer analyzed, as shown in Table 4.

Table 5 presents an estimate of the mean intake of phenolic compounds when an individual consumes a can (350 mL) of Brazilian craft beer, which were analyzed in this study.

## 3. Discussion

In general, the phenolic composition of the craft beer samples presented a varied concentration of the compounds according to the type of beer analyzed. Catechin showed higher concentrations in the analyzed samples, with a concentration of up to 124.8 mg L^−1^, followed by epicatechin (up to 51.1 mg L^−1^) and caffeic acid (up to 8.13 mg L^−1^) (Table 4).

Rutin was present in all Brazilian craft beer samples (0.39 to 2.94 mg L^−1^), while it was not observed in Brazilian industrial beers in previous works [5]. However, this same phenolic compound had already been observed in commercial beers analyzed in India. Rutin is a flavonoid found in buckwheat, a raw material widely used in the production of craft beers. In recent years, considering the importance of the physiological effects of rutin (anti-inflammatory activity, anti-arthritic, and antifungal effects in addition to helping to control some cancers, contributing to some neurological disorders, among others), buckwheat has been used as a substitute for other grains in the production of different types of beers [34,35,36,37].

Traditionally, beer is made from four basic ingredients: barley, hops, yeast, and water. The first two ingredients contain naturally phenolic compounds, enabling the incorporation of these substances to the beverage. However, variations in beer production methods, especially for craft beers, considerably influence the yield and phenolic profile of the beverage. Therefore, different combinations of ingredients in different proportions directly influence the phenolic composition of craft beers besides the type of barley and hops used for production. Briefly, the technological process of craft beers production (including genetic factors of the yeasts used in the fermentation process, conditions for growing raw materials of plant origin, among others) can result in a change in the phenolic profile of the beverage. The final content of phenolic compounds in craft beers depends on the selection of raw materials and the wort-production technology [2,8].

Considering the presented results, it is notable that samples 7 to 11 presented the highest total phenolic concentration (sum of the individual phenolic concentrations of each compound) (between 52.6 mg L^−1^ and 138.1 mg L^−1^) (Table 4). It is noteworthy that sample 7 has coffee in its composition, sample 9 shows addition of vanilla flavor, and sample number 11 is a mixed alcoholic beverage based on beer and honey. There are many studies in the literature that show that plant origin products (such as coffee and vanilla) and honey have a significant concentration of phenolic compounds in their composition, and our results show that the addition of these natural components in the beverage can contribute significantly to the phenolic composition of craft beer [38,39,40,41,42].

In a recent review, Ambra et al. (2021) [2] mentioned that fruit beers or beers added to fruit (and/or others plant origin products) during their production may be enriched with bioactive compounds that are undetectable in conventional beers, such as catechin, rutin, myricetin, quercetin, and resveratrol. Therefore, we emphasize that the production of craft beers involves the addition of products of natural origin, as already mentioned, and this can promote a significant enrichment in the composition and phenolic diversity of the beverage. In addition, generally, for producing craft beers, the base, raw materials are barley and hops and, less often, wheat, which may also contribute to the presence of different types of phenolic compounds in craft beers as well as an abundance of these bioactive compounds in the beverage [21].

Ulloa et al. (2017) [20] evaluated the influence of the addition of ethanolic extract of propolis at different concentrations on beer and observed an increase of up to 27% in the total phenolic composition and up to 59% in the flavonoid content of the beverage. Besides that, the addition of the propolis extract contributed to increase the antioxidant activity of the beer without altering the physicochemical parameters of the beverage.

The effect on phenolic-compounds content and sensorial properties in beers enriched with goji berries had been studied. The addition of goji berries increased substantially the content of phenolic acids in the beverage as well as resulting in a significant increase in antioxidant capacity of beer [11].

Similarly, Gasinski et al. (2020) [43] evaluated the possibility of using mango in beer-production technology. The authors observed that mango-added beers had polyphenol content up to 44% higher than the control beer as well as a significant increase in antioxidant activity compared to the control beer.

In a study with Italian special beers, Nardini and Foddai (2020) [25] verified that the highest antioxidant activities were obtained for walnut beer followed by cocoa, chestnut, licorice, and coffee beers. This fact is in accordance with the higher polyphenols and flavonoids total contents observed in these same samples.

Among the compounds studied in our work, formononetin was observed in all analyzed samples, with concentrations varying from 0.52 mg L^−1^ to 2.40 mg L^−1^. In contrast, kaempferol was the compound that was less present in the samples and was identified in only two Brazilian craft beers analyzed at concentrations below the limit of quantification. The levels of catechin (0.41 mg L^−1^–124.8 mg L^−1^) and epicatechin (1.43 mg L^−1^–51.1 mg L^−1^) found in Brazilian craft beers were higher than in commercial Chinese beers that used liquid–liquid extraction before analyzing the beer samples [44].

In general, most of the samples studied presented phenolic compounds with concentrations close to those obtained for industrial beers [5]. However, compounds such as ferulic acid were found in Brazilian craft beer in higher concentration than in Brazilian industrial beer, showing that these compounds can be from raw material (mainly natural products) used for production of craft beer. Similarly to our study, ferulic acid was reported as most abundant phenolic compound in European and Chinese beers, as quoted by Moura-Nunes et al. (2016) [5]. Wannenmacher et al. (2018) [29] mentioned that most studies describe ferulic and *p*-coumaric acids as the most abundant phenolic acids in beer.

Although there is a significant number of studies regarding the determination of phenolic compounds in beers, there are few that present a systematic study of the analytical method, presenting the figures of merit (such as limits of detection and quantification) or the method validation step. The method proposed in this work made it possible to evaluate the presence of phenolic compounds in Brazilian craft beers without using a previous step of extraction and/or pre-concentration of the analytes. The optimization of wavelengths for the compounds analyzed significantly contributed to obtaining a good sensitivity of the method, even performing direct injection of the craft beer samples. Our method showed a good sensitivity since the limits of detection (0.08–0.83 mg L^−1^) and limits of quantification (0.27–2.78 mg L^−1^) were close to those obtained in studies that used different sample-preparation techniques (such as SPE and LLE) associated with analysis techniques, such as HPLC-DAD or LC-MS, for the determination of phenolic compounds in beers (Table 6).

The mean intake of total phenolic compounds by Brazilian population is estimated at 460.15 mg per day. Diverse beverages are among the main foods that contribute to this consumption [33]. Considering the consumption of a can of Brazilian craft beer (350 mL), we can estimate that the individual would be ingesting between 0.97 mg and 48.3 mg of total phenolic compounds present in the samples analyzed in our study, which represents up to 11% of the total daily consumption of phenolic compounds by the Brazilian population (Table 5). It is evident that a higher intake of phenolic compounds occurs in craft beers, which present addition of vegetable products during the beverage-production process due to the incorporation of a greater amount of compounds from different origins to the final product.

## 4. Materials and Methods

### 4.1. Standards and Samples

The standards used for analysis of phenolic compounds were catechin, caffeic acid, epicatechin, *p*-coumaric acid, hydrated rutin, *trans*-ferulic acid, quercetin, kaempferol, and formononetin, which have already been identified in beers or could originate from plant products that are often added to craft beers. All standards were acquired from Sigma-Aldrich, with exception of *trans*-ferulic acid that was acquired from Fluka.

The solvents were methanol (JT Baker), acetic acid (Dinâmica), ultrapure water obtained from a Milli-Q system, and ethanol (JT Baker); all solvents were of HPLC grade.

A total of 14 samples of Brazilian craft beers and 1 sample of a mixed alcoholic beverage were acquired in commercial establishments in the city of Salvador-BA (Brazil). The types of craft beers studied were: Light Wheat Beer Weiss Type (sample 1), Extra Beer Pale Ale Type (sample 2), Extra Beer Vienna Lager Type (sample 3), Dark Strong Beer India Pale Ale Type (sample 4), Light Beer Pilsen (sample 5), Extra Wheat Beer With Cilantro and Orange Flavor (sample 6), Dark Strong Beer Porter With Coffee (sample 7), Dark Strong Beer Bock Type (sample 8), Dark Strong Beer Ale Type With Vanilla Flavor (sample 9), Pilsen Beer (sample 10), Imperial Strong Beer I.P.A Type (sample 12), Light Pure Malt Beer (sample 13), Pure Malt Beer With Passion Fruit American IPA Type (sample 14), and Pure Malt Dark Strong Beer Double Red Type (sample 15). The mixed alcoholic beverage analyzed was composed of beer and honey (sample 11). All samples were stored under refrigeration until analysis.

### 4.2. Analysis

The analyses of the phenolic compounds were done using a High Performance Liquid Chromatograph with Diode Array Detector (HPLC-DAD) (Shimadzu) equipped with an unit of quaternary solvent pumping (LC-20AT), an automatic injector (SIL-20AHT), degasser (DGU-205), furnace for column (CTO-20A), and a controller interface (CBM-20A). The chromatographic separation was performed using a NUCLEODUR^®^ 100-5 C18 ec (150 mm × 4 mm ID, 5 μm particle size) column coupled to a ZORBAX Eclipse Plus C18 Agilent (4.6 mm × 12.5 mm) pre-column.

Acetic acid 5% (solvent A) and acetonitrile (solvent B) were used as mobile phase, which had varying percentages throughout the analysis: 0 to 35 min (0–92% B); 35 to 40 min (92–0% B); and 40 to 42 min (0% B). All runs had a total time of 42 min at a constant flow of 1.00 mL min^−1^ and oven temperature of 40 °C.

**Figure 3:**
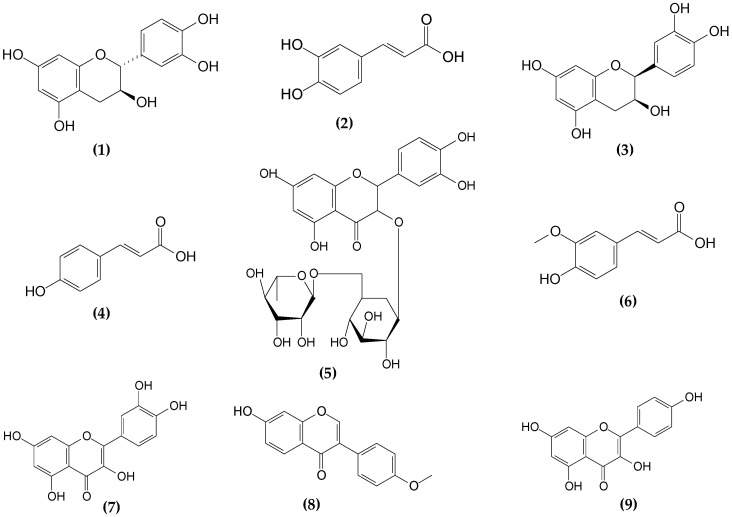
Structural formulas of phenolic compounds analyzed in Brazilian craft beers. (**1**) Catechin, (**2**) caffeic acid, (**3**) epicatechin, (**4**) *p*-coumaric acid, (**5**) rutin, (**6**) *trans*-ferulic acid, (**7**) quercetin, (**8**) kaempferol, and (**9**) formononetin.

Nine phenolic compounds were analyzed (catechin, caffeic acid, epicatechin, *p*-coumaric acid, hydrated rutin, *trans*-ferulic acid, quercetin, kaempferol, and formonometin) (Figure 3), and the analytical curves, for the quantification of the analytes, were constructed from a mixture of all compounds at a concentration of 40 mg L^−1^ (stock solution) in methanol. By means of dilutions of the stock solution, the following concentrations were prepared for the respective analytical curves in 5% ethanol (for simulating the alcoholic strength of beer samples): 0.1 mg L^−1^; 0.25 mg L^−1^; 0.50 mg L^−1^; 1.0 mg L^−1^; 5.0 mg L^−1^; 7.5 mg L^−1^; 10.0 mg L^−1^; and 12.5 mg L^−1^.

In order to determine the concentration of phenolic compounds in craft beers, the chromatographic analyses were preceded by the degassing of the samples by sonication followed by filtering (regenerated cellulose, 0.45 μm). The injected volume of each sample and standard solutions was 20 μL and was analyzed in triplicate.

#### Quality Assurance and Quality Control (QA/QC)

To ensure good quality of the analytical results and to avoid contamination, all glassware was washed using alcoholic potash solution, Extran^®^ detergent, and ultrapure water, sequentially. In addition, the bottles containing the craft beers samples purchased in commercial establishments were opened only at the time of analysis.

We performed analyses of the equipment blanks and solvent blanks for assessing the presence of possible interfering peaks in the chromatograms.

After the analysis, the chromatographic column was systematically washed with ultrapure water and organic solvent (acetonitrile). Then, to store the column, it was filled with organic solvent to avoid degradation of the stationary phase.

### 4.3. Optimization of Wavelengths for the Phenolic Compounds Detection

After evaluating the UV-vis spectra of each standard solution individually, the wavelengths of higher absorption of the UV-vis radiation were selected in order to obtain greater sensitivity of the determination of the phenolic compounds in the craft beer samples. Therefore, the choice of the optimum wavelength for each analyte was essential so that it was not necessary to use an extraction method for the analysis of the phenolic compounds in the craft beers. It is noteworthy that the works that determine phenolic compounds in different matrices used a single wavelength, typically at 280 nm, for the detection of these compounds [5,47,48].

**Figure 4:**
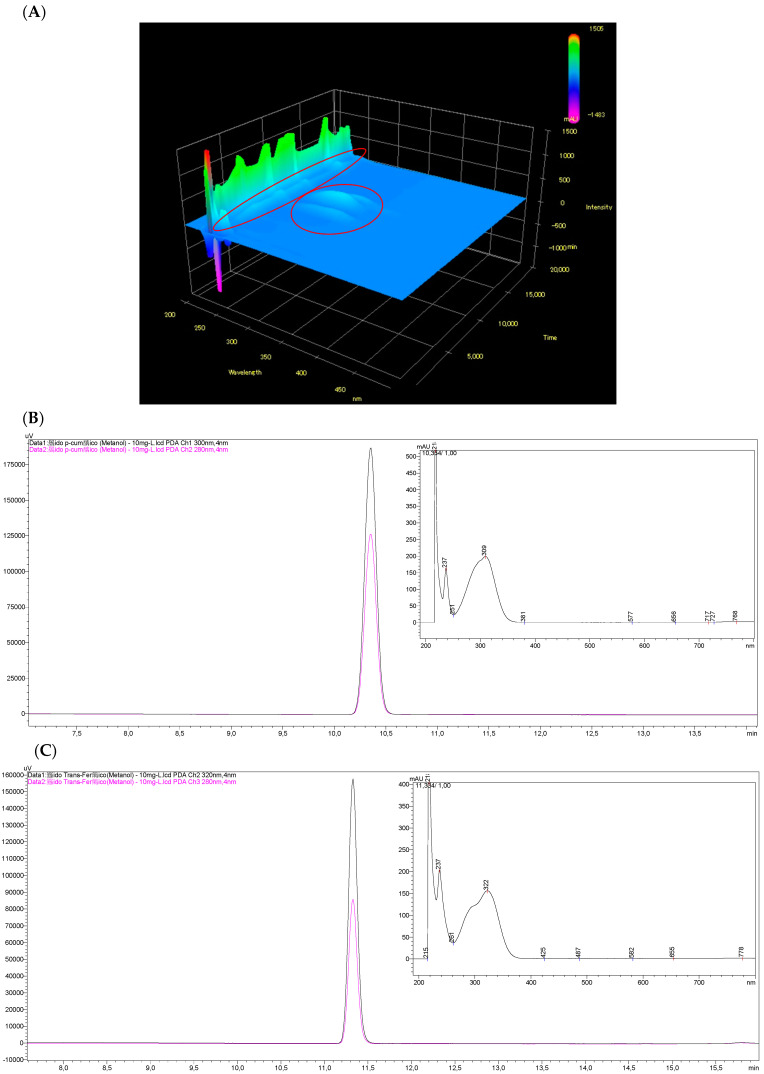
(**A**) 3D spectrum for a standard solution of phenolic compounds (12.5 mg L^−1^); chromatograms and UV-vis spectra obtained for (**B**) *p*-coumaric acid (10 mg L^−1^) and (**C**) *trans*-ferulic acid (10 mg L^−1^), individually injected, for the choice of the wavelength of higher absorption of the electromagnetic radiation in the diode array detector (in the chromatograms: pink = 280 nm; black = 320 nm for *p*-coumaric acid; and 300 nm for *trans*-ferulic acid).

The 3D spectrum clearly shows two wavelength regions (close to 280 nm and in the range from 300 to 350 nm, highlighted in the graph with red circles) with greater intensity of electromagnetic radiation absorption by the analyzed compounds, especially for analytes with retention times between 7.5 and 15.0 min (Figure 4). Thus, the individual spectra of the analyzed phenolic compounds were evaluated.

Figure 4 presents the UV-vis spectra for some of the phenolic compounds analyzed in the craft beer samples for which it was possible to obtain the wavelength of greater absorption of the electromagnetic radiation. Chromatograms and spectra of the other phenolic compounds analyzed in our study are presented in the Supplementary Material (Figures S1 and S2). After that, it was possible to choose the following wavelengths for the analysis of phenolic compounds in craft beers: 280 nm (catechin and epicatechin), 300 nm (caffeic acid, *p*-coumaric acid, and formononetin) and 320 nm (hydrated rutin, *trans*-ferulic acid, quercetin, and kaempferol).

It is possible to observe a significant increase in the peak area for the *p*-coumaric acid (320 nm) and the *trans*-ferulic acid (300 nm) when a wavelength of greater electromagnetic radiation absorption was selected compared to wavelength of 280 nm, typically used in the chromatographic analysis of phenolic compounds in different matrices. Thus, the sensitivity required for the analysis of phenolic compounds in craft beers was obtained without the need for an extraction stage of the analytes.

### 4.4. Validation of the Method

In order to ensure the analytical quality of the results, procedures were performed to validate the method in which the following parameters were evaluated: selectivity, linearity, limit of detection, limit of quantification, precision, and accuracy [32,49].

The limits of detection and quantification were obtained using the parameters of the analytical curves constructed and calculated by their mathematical relationships: LOD = 3SD/*m*, and LOQ = 10SD/*m* (where SD = estimative of the standard deviation of the regression line, and m = angular coefficient of the calibration line).

For the evaluation of intraday precision, five different solutions were prepared with the mixture of all phenolic compounds at three concentration levels (0.5 mg L^−1^, 7.5 mg L^−1^, and 12.5 mg L^−1^). All these solutions were prepared by dilutions from stock solution and were then injected in triplicate into the chromatographic system. The precision of the analytical method was evaluated in relation to the levels of repeatability, estimating the relative standard deviation (RSD) for each compound analyzed from successive measurements.

We also performed tests to evaluate the accuracy of the method, which represents the degree of agreement between the data obtained in a given analysis and a previously known value. This step was performed by means of recovery tests by standard addition at three concentration levels (0.5 mg L^−1^, 7.5 mg L^−1^, and 12.5 mg L^−1^) in one of the real samples of craft beer. After the analyses in triplicate of each of the solutions prepared, the recovery was calculated by the following mathematical equation:

%Recovery = [measured concentration/expected concentration] × 100(1)

## 5. Conclusions

By means of a simple and fast method, it was possible to identify and quantify phenolic compounds in craft beers without extraction of the analytes and obtaining good sensitivity by performing the detection of the compounds at different wavelengths. Direct injections of the samples were performed in the chromatographic system without the use of organic solvents in the preparation step of the samples, which could be in accordance to principles of Green Analytical Chemistry.

The optimization of wavelengths for the detection of phenolic compounds using the liquid chromatography with diode array detection (HPLC-DAD) can be an essential step for improving the sensitivity of an analytical method in the analysis of beverages or other matrices.

Rutin and formononetin were present in all samples of craft beers analyzed. However, catechin, epicatechin, and caffeic acid were the phenolic compounds that presented the highest concentrations in Brazilian craft beers. The presence of plant origin products, such as coffee, vanilla and honey, was the determinant for the occurrence of the highest concentrations of phenolic compounds in Brazilian craft beers.

## Figures and Tables

**Table 1:** Figures of merit obtained for the analysis of phenolic compounds in craft beers.

Phenolic Compounds	Linear Range (mg L^−1^)	R^2^	LOD * (mg L^−1^)	LOQ * (mg L^−1^)
Catechin	0.10–12.5	0.9999	0.08	0.28
Caffeic acid	0.25–12.5	0.9994	0.22	0.74
Epicatechin	0.50–12.5	0.9980	0.39	1.29
*p*-Coumaric acid	0.10–10.0	0.9999	0.08	0.27
Hydrated rutin	0.25–12.5	0.9999	0.10	0.35
*Trans*-ferulic acid	0.10–10.0	0.9998	0.08	0.28
Quercetin	0.50–12.5	0.9990	0.35	1.16
Kaempferol	1.0–12.5	0.9966	0.83	2.78
Formononetin	0.10–7.5	0.9997	0.09	0.32

* LOD, limit of detection; LOQ, limit of quantification.

**Table 2:** Relative standard deviations (RSD) obtained for each phenolic compound in the evaluation of method repeatability (intraday precision).

Phenolic Compounds	RSD (%) *		
	0.5 mg L^−1^	7.5 mg L^−1^	12.5 mg L^−1^
Catechin	4.20	5.70	2.77
Caffeic acid	4.51	4.50	3.08
Epicatechin	5.19	5.22	3.85
*p*-Coumaric acid	5.19	5.21	3.73
Hydrated rutin	14.5	11.5	16.2
*Trans*-ferulic acid	6.78	4.53	5.86
Quercetin	7.68	4.76	2.75
Kaempferol	5.72	3.38	2.59
Formononetin	4.68	5.30	3.15

* RSD, relative standard deviation for five different preparations of the standard solution in three levels of concentration, injected on the same day in triplicate (*n* = 15).

**Table 3:** Accuracy (recovery tests) of the method for determination of phenolic compounds in Brazilian craft beers.

Phenolic Compounds	Concentration in the Sample * (mg L^−1^)	Added Concentration (mg L^−1^)		
		0.5	7.5	12.5
			Accuracy (%)	
Catechin	ND	105	103	95.9
Caffeic acid	ND	85.1	85.8	93.9
Epicatechin	1.43	88.6	94.3	99.3
*p*-Coumaric acid	ND	87.5	86.4	88.2
Hydrated rutin	0.61	112	88.3	91.4
*Trans*-ferulic acid	<LOQ	111	90.9	94.2
Quercetin	<LOQ	91.8	95.1	105
Kaempferol	<LOQ	69.8	98.2	99.2
Formononetin	0.70	68.6	101	105

* ND, not detected; LOQ, limit of quantification.

**Table 4:** Concentration (mg L^−1^) of the phenolic compounds in Brazilian craft beers samples.

**Phenolic Compounds**	Concentration (mg L^−1^) *							
	**Sample 1**	**Sample 2**	**Sample 3**	**Sample 4**	**Sample 5**	**Sample 6**	**Sample 7**	**Sample 8**
Catechin	ND	ND	ND	16.8 ± 0.3	0.41 ± 0.02	ND	38.6 ± 0.08	65.2 ± 1.6
Caffeic acid	ND	ND	ND	<LOQ	<LOQ	<LOQ	8.13 ± 0.21	ND
Epicatechin	1.43 ± 0.07	2.50 ± 0.16	3.16 ± 0.22	3.55 ± 0.07	1.94 ± 0.08	ND	51.1 ± 0.0	ND
*p*-Coumaric acid	ND	0.97 ± 0.02	1.11 ± 0.04	ND	0.70 ± 0.01	<LOQ	0.47 ± 0.06	ND
Hydrated rutin	0.62 ± 0.02	0.92 ± 0.02	0.62 ± 0.02	1.07 ± 0.02	0.39 ± 0.02	0.93 ± 0.03	1.45 ± 0.04	1.13 ± 0.04
*Trans*-ferulic acid	<LOQ	2.10 ± 0.03	2.19 ± 0.01	1.74 ± 0.02	1.57 ± 0.04	1.42 ± 0.02	1.34 ± 0.11	1.59 ± 0.13
Quercetin	<LOQ	<LOQ	<LOQ	<LOQ	<LOQ	<LOQ	ND	<LOQ
Kaempferol	<LOQ	<LOQ	ND	ND	ND	ND	ND	ND
Formononetin	0.71 ± 0.02	1.47 ± 0.01	1.55 ± 0.02	1.70 ± 0.02	1.88 ± 0.01	1.49 ± 0.00	2.40 ± 0.06	0.68 ± 0.04
**Total (Sum of the phenolic compounds)**	2.76	7.96	8.63	24.9	6.89	3.84	103.5	68.6
**Phenolic Compounds**		Concentration (mg L^−1^) *						
	**Sample 9**	**Sample 10**	**Sample 11**	**Sample 12**	**Sample 13**	**Sample 14**	**Sample 15**	
Catechin	37.4 ± 0.9	108.3 ± 1.7	124.8 ± 1.9	ND	ND	ND	ND	
Caffeic acid	<LOQ	<LOQ	<LOQ	<LOQ	<LOQ	<LOQ	<LOQ	
Epicatechin	6.73 ± 0.36	ND	9.12 ± 0.28	ND	2.46 ± 0.09	2.61 ± 0.06	ND	
*p*-Coumaric acid	4.17 ± 0.23	<LOQ	ND	<LOQ	0.66 ± 0.00	<LOQ	<LOQ	
Hydrated rutin	1.36 ± 0.02	0.79 ± 0.02	1.17 ± 0.09	2.94 ± 0.04	1.29 ± 0.02	0.63 ± 0.08	0.49 ± 0.02	
*Trans*-ferulic acid	1.26 ± 0.09	0.89 ± 0.02	1.31 ± 0.06	<LOQ	<LOQ	<LOQ	0.66 ± 0.01	
Quercetin	1.19 ± 0.02	<LOQ	<LOQ	<LOQ	<LOQ	<LOQ	1.45 ± 0.02	
Kaempferol	ND	ND	ND	ND	ND	ND	<LOQ	
Formononetin	0.52 ± 0.01	0.74 ± 0.01	1.67 ± 0.00	1.88 ± 0.01	1.35 ± 0.02	1.08 ± 0.02	1.67 ± 0.04	
**Total (Sum of the phenolic compounds)**	52.6	110.7	138.1	4.82	5.76	4.32	4.27	

* Mean ± standard deviation; ND, not detected; <LOQ, below the limit of quantification.

**Table 5:** Estimation of total phenolic compounds intake in a can (350 mL) of Brazilian craft beer.

	**Samples**							
	**1**	**2**	**3**	**4**	**5**	**6**	**7**	**8**
**Total phenolic compounds (mg per drink) ^a^**	0.97	2.79	3.02	8.72	2.41	1.34	36.2	24.0
**Daily value (%) ^b^**	0.2	0.6	0.7	1.9	0.5	0.3	7.9	5.2
	**Samples**							
	**9**	**10**	**11**	**12**	**13**	**14**	**15**	
**Total phenolic compounds (mg per drink) ^a^**	18.4	38.7	48.3	1.69	2.02	1.51	1.49	
**Daily value (%) ^b^**	4.0	8.4	11	0.4	0.4	0.3	0.3	

^a^ A drink = a can of 350 mL; ^b^ Based on the mean daily intake of total phenolic compounds by Brazilian population (460.15 mg per day) [33].

**Table 6:** Sample-preparation techniques used to analyze phenolic compounds in beers.

Compounds Analyzed	Sample-Preparation Technique	Analysis Technique	LOD (mg L^−1^)	LOQ (mg L^−1^)	Concentration Range (mg L^−1^)	Ref.
Gallic acid; protocatechuic acid; gentisic acid; catechin; caffeic acid; epicatechin; *p*-coumaric acid; ferulic acid; salicylic acid; quercetin	Solid-phase extraction (SPE)	HPLC-DAD	0.04–0.41	0.14–0.80	0.28–5.04	[10]
Gallic acid; (-)-catechin; epicatechin; ferulic acid; chlorogenic acid; morin; rutin; quercetin; kaempferol; naringenin; luteolin	Liquid–liquid extraction (LLE)	LC-PDA LC-ESI-MS	0.02–0.04 0.006–0.012	0.06–0.012 0.02–0.04	0.24–5.70 0.010–2.38	[45]
Gallic acid; vanillic acid; *p*-coumaric acid; *trans*-ferulic acid; sinapic acid; caffeic acid; syringic acid; 4-hydroxibenzoic acid; (+)-catechin; hydrate quercetin; 3-caffeoylquinic acid; xanthohumol; isoxanthohumol; 8-prenylnarigenin; cohumulone, colupulone; lupulone; colupulone	Dilution Solid-phase extraction (SPE) Simplified liquid extraction (SLE)	HPLC-API-MS/MS	0.001–0.150	0.004–0.500	0.1–34.4 (µg kg^−1^)	[46]
Catechin; caffeic acid; epicatechin; *p*-coumaric acid; hydrated rutin; *trans*-ferulic acid; quercetin; kaempferol and formononetin	Not applicable	HPLC-DAD	0.08–0.83	0.27–2.78	<0.27–124.8	This work

## Data Availability

The data presented in this study are available on request from the corresponding author. The data are not publicly available due to the authors’ ownership of the results.

## Supplementary Materials

The following are available online, Figure S1. UV-vis spectra obtained for (A) catechin, (B) caffeic acid, (C) epicatechin, (D) rutin, (E) quercetin, (F) kaempferol, and (G) formononetin injected at the concentration of 10 mg L^−1^ in the HPLC-DAD for the choice of the wavelength of higher absorption of the electromagnetic radiation in the diode array detector. The spectra of compounds *p*-coumaric acid and *trans*-ferulic acid are shown in the main manuscript; Figure S2. Chromatograms obtained by HPLC-DAD for 1. caffeic acid; 2. hydrated rutin; 3. *trans*-ferulic acid; 4. quercetin; 5. kaempferol; and 6. formononetin injected at the concentration of 10 mg L^−1^ for the choice of the wavelength of higher absorption of the electromagnetic radiation in the diode array detector (pink = 280 nm; blue = 300 nm; and brown = 320 nm). The chromatograms of the compounds catechin and epicatechin were not shown as they were analyzed at the wavelength typically used (280 nm) for the analysis of phenolic compounds in different matrices in according to the spectra showed in Figure S1. The chromatogram of the *p*-coumaric acid is shown in the main manuscript; Figure S3. Chromatogram of the same craft beer sample presented in the main manuscript (Figure 1B), with the superposition of the different wavelengths used in the analysis of phenolic compounds. The identification of the phenolic compounds in the chromatogram was made according to the respective optimized wavelengths: 1. catechin; 2. caffeic acid; 3. epicatechin; 5. hydrated rutin; 6. *trans*-ferulic acid; 7. quercetin; and 9. formononetin (green = 280 nm; pink = 300 nm; and blue = 320 nm).

## Sample Availability

Samples of the compounds are not available from the authors.
