# Supplementary material for: A Simple Method for Evaluating the Bioactive Phenolic Compounds’ Presence in Brazilian Craft Beers
Source: Molecules. 2021 Aug 4;26(16):4716. doi: 10.3390/molecules26164716 (PMC8399525; doi:10.3390/molecules26164716)
Supplement: Supplementary file 1 [file molecules-26-04716-s001.zip › molecules-1302339-supplementary.pdf]

molecules

MDPI

A Simple Method for Evaluating the Bioactive Phenolic Com-pounds' Presence in Brazilian Craft Beers

Marcelo Coelho Silva [1], Jeancarlo Pereira dos Anjos [1,2,*], Lilian Lefol Nani Guarieiro [1,2] and Bruna A. Souza Machado [1]

[1] Centro Universitário SENAI CIMATEC, Avenida Orlando Gomes, 1845-Piatã, Salvador 41650-010, BA, Brazil; celo12br@hotmail.com (M.C.S.); lilian.guarieiro@fieb.org.br (L.L.N.G.); brunam@fieb.org.br (B.A.S.M.)
[2] INCT de Energia e Ambiente, UFBA, Salvador 40170-290, BA, Brazil
* Correspondence: jeancarlopanjos@gmail.com; Tel.: +55-71-3879-5677

Supplementary Material

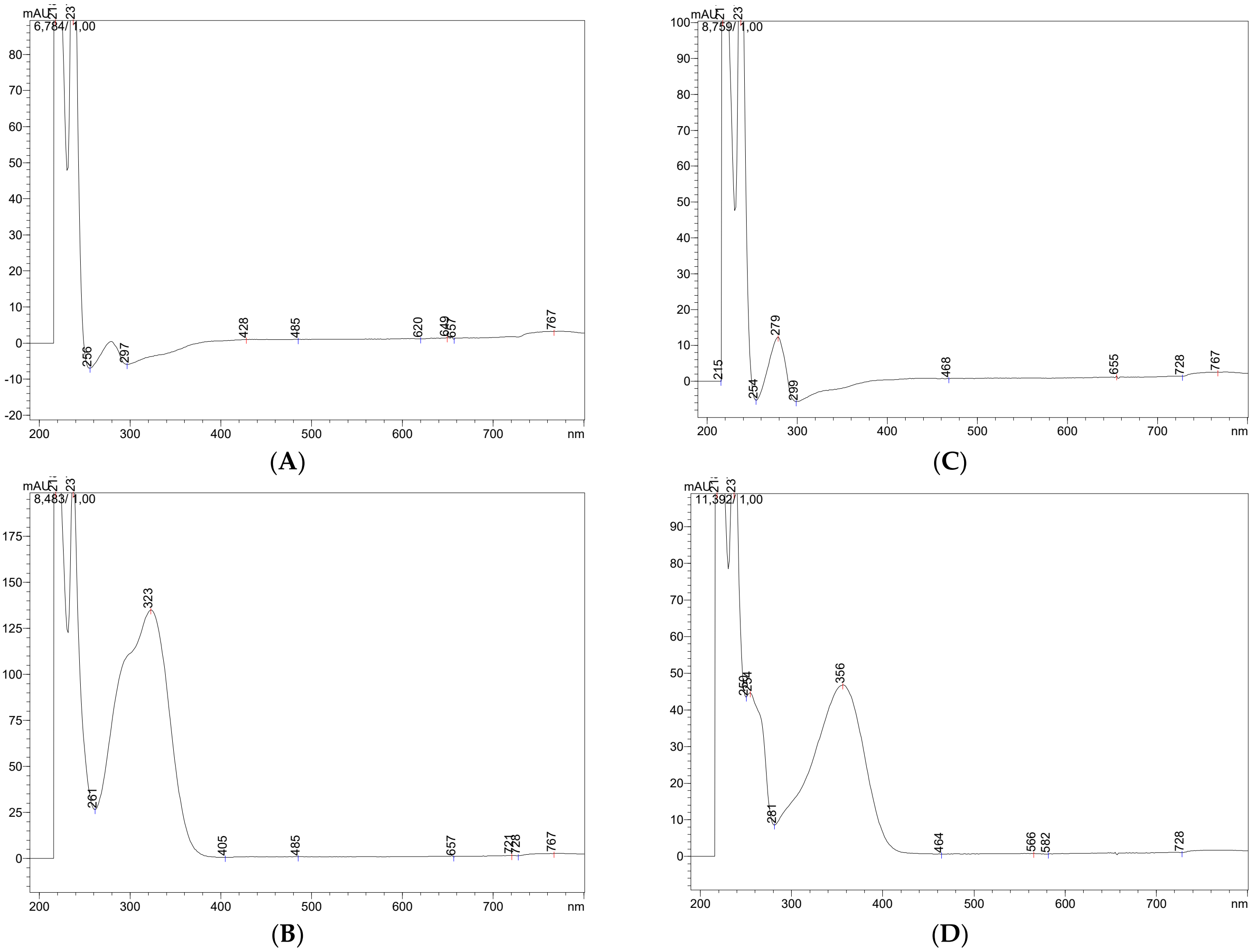

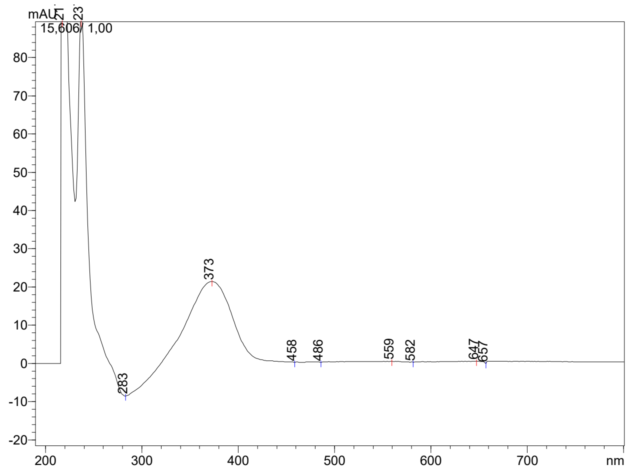

(**E**)

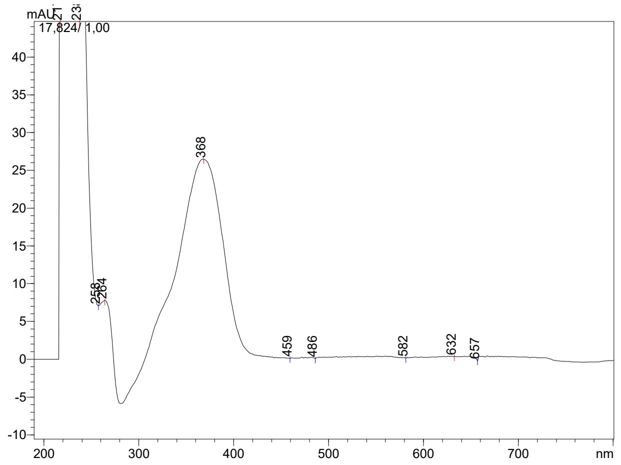

(**F**)

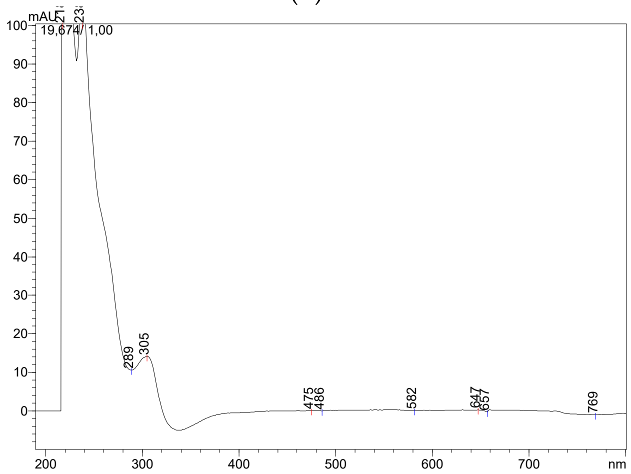

(**G**)

Figure S1. UV-vis spectra obtained for (A) catechin, (B) caffeic acid, (C) epicatechin, (D) rutin, (E) quercetin, (F) kaempferol and (G) formononetin, injected at the concentration of 10 mg L^{-1} in the HPLC-DAD, for the choice of the wavelength of higher absorption of the electromagnetic radiation in the diode array detector. The spectra of compounds *p*-coumaric acid and *trans*-ferulic acid are shown in the main manuscript.

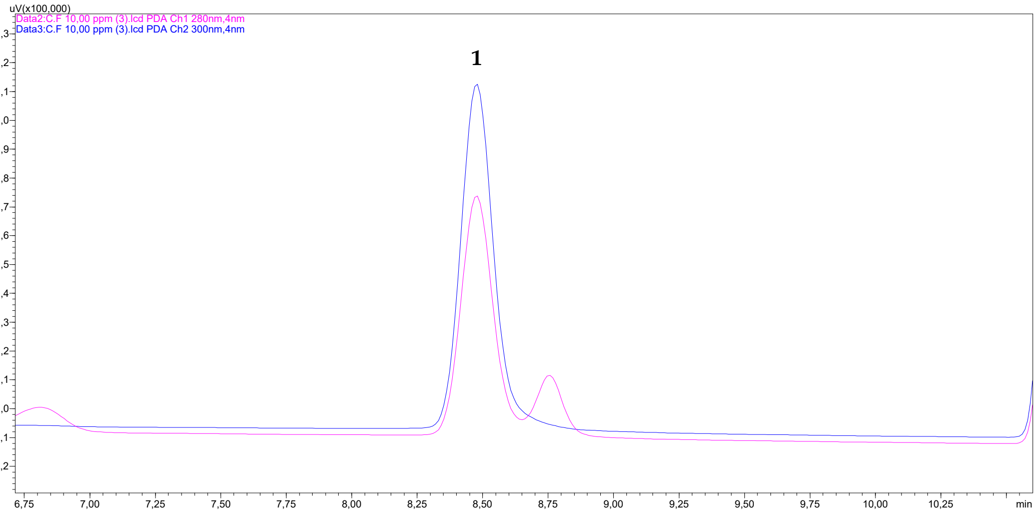
uV(x100,000)
Data2:C.F 10,00 ppm (3).lcd PDA Ch1 280nm,4nm
Data3:C.F 10,00 ppm (3).lcd PDA Ch2 300nm,4nm
1
min

(**A**)

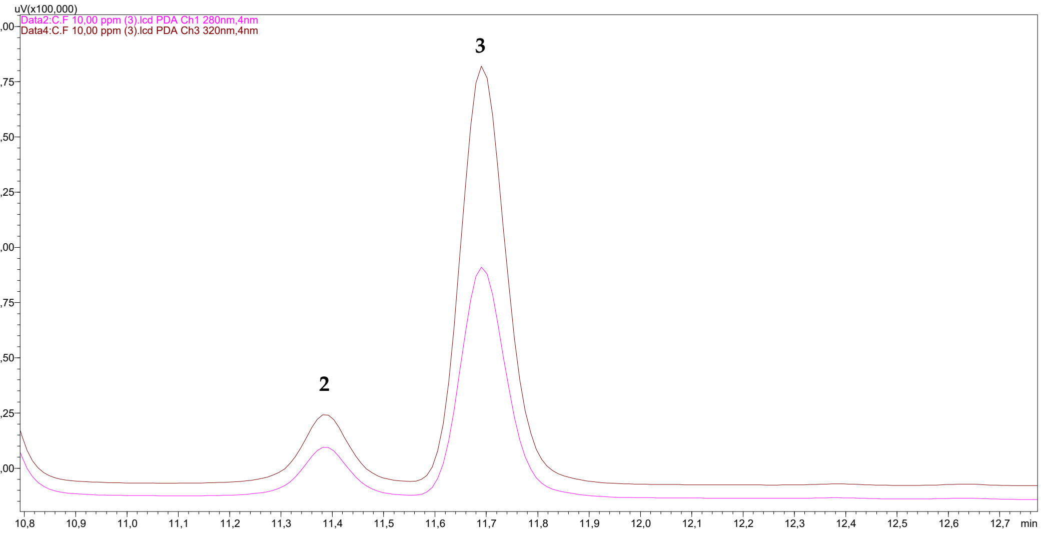
uV(x100,000)
Data2:C.F 10,00 ppm (3).lcd PDA Ch1 280nm,4nm
Data4:C.F 10,00 ppm (3).lcd PDA Ch3 320nm,4nm
2
3
min

(**B**)

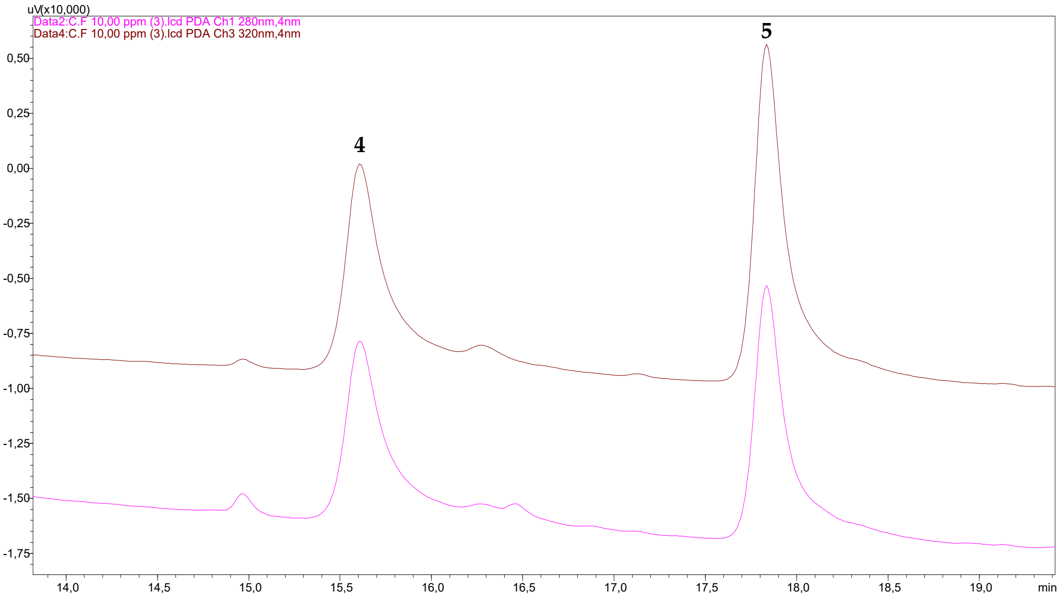
uV(x10,000)
Data2:C.F 10,00 ppm (3).lcd PDA Ch1 280nm,4nm
Data4:C.F 10,00 ppm (3).lcd PDA Ch3 320nm,4nm
4
5
min

(**C**)

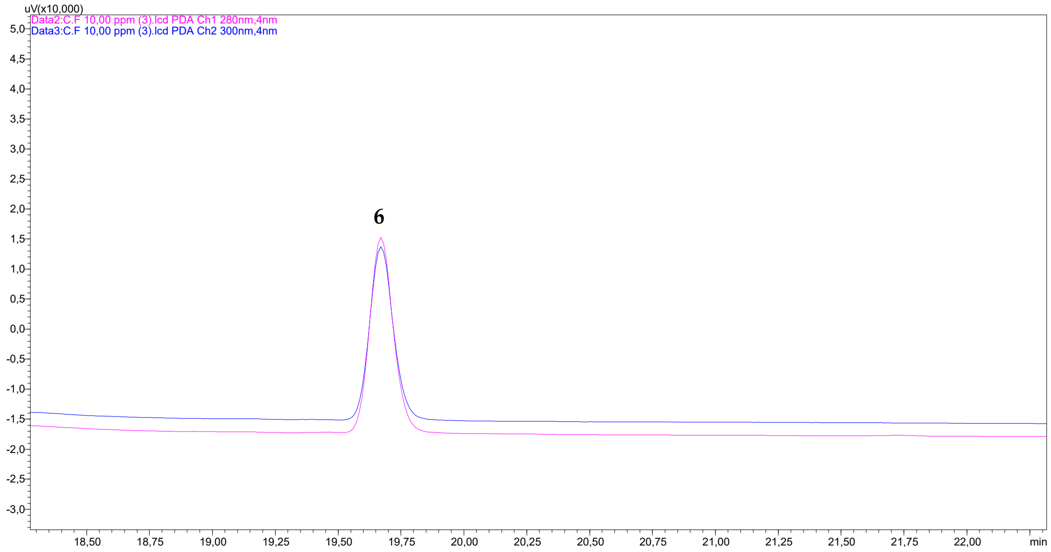

(**D**)

Figure S2. Chromatograms obtained by HPLC-DAD for 1. caffeic acid; 2. hydrated rutin; 3. *trans*-ferulic acid; 4. quercetin; 5. kaempferol; 6. formononetin, injected at the concentration of 10 mg L^{-1}, for the choice of the wavelength of higher absorption of the electromagnetic radiation in the diode array detector (Pink = 280 nm; Blue = 300 nm and Brown = 320 nm). The chromatograms of the compounds catechin and epicatechin were not shown as they were analyzed at the wavelength typically used (280 nm) for the analysis of phenolic compounds in different matrices, in according to the spectra showed in Figure S1. The chromatogram of the *p*-coumaric acid is shown in the main manuscript.

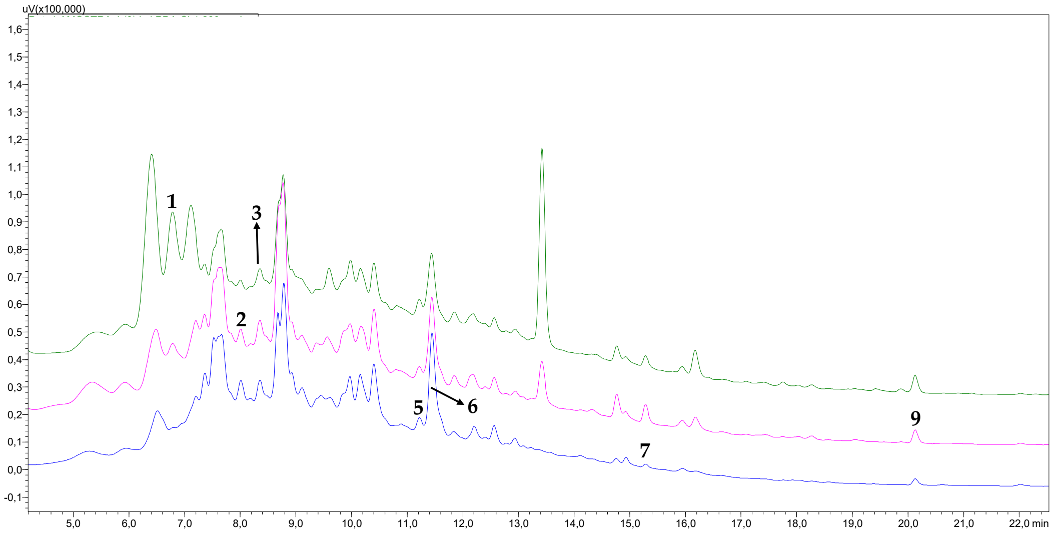

Figure S3. Chromatogram of the same craft beer sample presented in the main manuscript (Figure 1B), with the superposition of the different wavelengths used in the analysis of phenolic compounds. The identification of the phenolic compounds in the chromatogram was made according to the respective optimized wavelengths: 1. catechin; 2. caffeic acid; 3. epicatechin; 5. hydrated rutin; 6. trans-ferulic acid; 7. quercetin; 9. formononetin. (Green = 280 nm; Pink = 300 nm and Blue = 320 nm). The compounds *p*-coumaric acid and kaempferol were not detected in this craft beer sample.
